# Lower Micronutrient Intake in Patients with Coronary Artery Disease: A Cross-Sectional Study

**DOI:** 10.3390/healthcare14162611

**Published:** 2026-08-19

**Authors:** Erika Martinez-Lopez, Mariana Perez-Robles, Sissi Godinez-Mora, Alondra Mora-Jiménez, Alejandra Muñoz-Hernandez, Daniela Nuñez-García, Sarai Citlalic Rodríguez-Reyes, Liliana Estefanía Ramos-Villalobos, Wendy Campos-Perez

**Affiliations:** 1 Instituto de Nutrigenética y Nutrigenómica Traslacional, Departamento de Biología Molecular y Genómica, Centro Universitario de Ciencias de la Salud, Universidad de Guadalajara, Guadalajara 44100, Jalisco, Mexico; erika.martinez@academicos.udg.mx (E.M.-L.); mariana.perez@academicos.udg.mx (M.P.-R.); sissi.godinez5582@alumnos.udg.mx (S.G.-M.); citlalic.rodriguez@academicos.udg.mx (S.C.R.-R.); 2 Doctorado en Ciencias en Biología Molecular en Medicina, Departamento de Biología Molecular y Genómica, Centro Universitario de Ciencias de la Salud, Universidad de Guadalajara, Guadalajara 44100, Jalisco, Mexico; alondra.mora2239@alumnos.udg.mx (A.M.-J.); cristina.munoz4799@alumnos.udg.mx (A.M.-H.); daniela.nunez5529@alumnos.udg.mx (D.N.-G.); 3 Servicio de Cardiología del Hospital Civil de Guadalajara “Dr. Juan I. Menchaca”, Guadalajara 44340, Jalisco, Mexico; liliana.ramos5375@academicos.udg.mx

**Keywords:** micronutrients, coronary artery disease, diet, antioxidants

## Abstract

**Background/Objectives:** Coronary artery disease (CAD) is influenced by diet; however, the role of micronutrients remains underexplored. This study aimed to evaluate differences in dietary micronutrient intake between patients with and without CAD. **Methods:** A cross-sectional study was conducted with 107 participants (51 CAD, 56 non-CAD). Dietary intake was assessed using habitual 24-h dietary recalls and analyzed for micronutrient content. Biochemical, anthropometric, and clinical data were also evaluated. **Results**: CAD patients exhibited lower adequate intake of vitamins A, C, and K, as well as antioxidants such as lycopene and lutein, compared to the non-CAD group. While micronutrient inadequacy was prevalent in both groups, it was significantly more pronounced in subjects with CAD. **Conclusions:** Subjects with CAD had lower micronutrient intake than those without CAD, highlighting the potential importance of adequate micronutrient intake in the prevention and management of CAD.

## 1. Introduction

Dietary patterns are central to the prevention and progression of cardiovascular diseases. While macronutrient composition, particularly the balance between omega-6 (n-6) and omega-3 (n-3) polyunsaturated fatty acids (PUFA), has been extensively associated with heart health, micronutrient intake has received less attention [1]. Micronutrients, including vitamins, minerals, and trace elements, serve as essential cofactors in antioxidant defense, endothelial function, lipid metabolism, and inflammatory regulation, key pathways in atherogenesis and vascular homeostasis. Consequently, inadequate intake of these nutrients may promote oxidative stress, endothelial dysfunction, and chronic inflammation, processes that are central to cardiovascular pathophysiology [2,3].

Recent studies suggest that low intake of vitamin D, magnesium, selenium, and vitamin K is associated with increased cardiovascular risk due to their roles in vascular calcification and endothelial homeostasis [4,5,6,7]. However, clinical trials evaluating supplementation with some of these micronutrients have not shown clear benefits on cardiovascular outcomes [8]. These findings suggest that cardiovascular risk may be more closely related to overall diet quality and nutritional status than to micronutrient supplementation in isolation.

The blood vessel obstruction by accumulation of fatty plaques that thicken and harden the arterial walls, a process known as atherosclerosis, is one of the main characteristics of CAD. This results in reduced blood and oxygen supply to the myocardium and can manifest clinically as stable angina, unstable angina, myocardial infarction (MI), or sudden cardiac death. Moreover, CAD continues to be a leading cause of death in both developed and developing countries [9,10].

The main risk factors for the development of CAD include age (>40 years), sex (with a higher prevalence in men), and the presence of comorbidities such as obesity, hypertension, dyslipidemias, modification and oxidation of plasma lipoproteins, type 2 diabetes mellitus (T2DM), insulin resistance, and increased waist circumference (WC). In addition, unhealthy habits such as an atherogenic diet, physical inactivity, alcohol consumption, and smoking are also associated with CAD development, as they exert a considerable influence on serum lipid concentrations and vascular wall integrity. Several genetic variants involved in lipid metabolism and homeostasis also contribute to disease susceptibility [1,11].

However, despite the well-established role of diet in modulating cardiovascular risk, relatively few studies have comprehensively assessed dietary micronutrient intake in patients with CAD, particularly in comparison with individuals without the disease. A clearer understanding of these nutritional differences could provide new insights into modifiable risk factors and identify potential targets for dietary interventions. Therefore, the aim of this study was to evaluate differences in dietary micronutrient intake between CAD and non-CAD individuals.

## 2. Materials and Methods

### 2.1. Study Subjects

A total of 107 participants were included: 51 with a diagnosis of CAD and 56 non-CAD. Participants were recruited using convenience sampling between February 2024 and December 2025. Invitations to participate were disseminated through social media and printed flyers displayed on bulletin boards at the Hospital Civil de Guadalajara “Dr. Juan I. Menchaca” and the Familiar Clinic No. 1 of the Instituto de Seguridad y Servicios Sociales de los Trabajadores del Estado (ISSSTE) “Dr. Arturo González Guzmán”. All participants were subsequently evaluated at the Instituto de Nutrigenética y Nutrigenómica Traslacional of the Universidad de Guadalajara. The inclusion criteria for subjects with CAD were a confirmed diagnosis within the previous two years and receipt of medical care at the cardiology departments of the participating hospitals. Non-CAD participants had no previous diagnosis of CAD and were recruited from those who responded to the recruitment invitation. For both groups, additional inclusion criteria were age between 30 and 75 years, body mass index (BMI) >18.5 kg/m^2^, no use of supplements, and no diagnosis of autoimmune diseases, cancer, or chronic kidney disease. Exclusion criteria for both groups were incomplete data and pregnancy or lactation.

### 2.2. Clinical Evaluation of Non-CAD Subjects

Non-CAD subjects were evaluated using the Rose Angina Questionnaire. Information was collected on chest pain characteristics, medication use, and history of coronary artery disease. Subjects with a history of acute myocardial infarction, angina pectoris, symptoms suggestive of angina, or prior coronary revascularization procedures were not included.

### 2.3. Diagnosis of CAD

CAD was diagnosed by a cardiologist based on the clinical evaluation of symptoms indicative of myocardial infarction, as previously described [12]. In men, the symptoms considered included oppressive chest pain radiating to the left arm, dyspnea, and retrosternal pain. In women, fatigue, balance disturbances, arrhythmias, and shortness of breath were considered. In addition, biomarkers of myocardial injury, including creatine kinase and cardiac troponins, were assessed. All patients exhibited ST-segment abnormalities and coronary obstruction, as confirmed by electrocardiography and coronary angiography, respectively.

### 2.4. Pharmacological Therapy

Subjects with CAD received standard medical therapy according to their clinical requirements. Pharmacological treatments included lipid-lowering agents (atorvastatin, bezafibrate, pravastatin, and ciprofibrate), vasodilators (isosorbide and nifedipine), antiplatelet drugs (acetylsalicylic acid and clopidogrel), antihypertensive drugs (metoprolol, losartan, and enalapril), and glucose-lowering therapy (metformin) when indicated.

### 2.5. Dietary Intake Assessment

Each subject was interviewed by a professional nutritionist. Dietary intake was assessed using a food recall based on a habitual day to obtain information on the types and quantities of food consumed and characterize the participants’ usual dietary patterns, as described in previous studies [13]. It was verified that the information collected did not correspond to an atypical day of food consumption. Dietary intake was then analyzed using Nutritionist Pro™ diet analysis software version 7.9 (Axxya Systems, Stafford, TX, USA).

For participants with CAD, the dietary assessment was intended to reflect habitual dietary intake at the time of diagnosis. Participants were asked whether they had modified their diet after diagnosis. If no dietary changes had occurred, their current habitual diet was recorded. If participants reported dietary changes after diagnosis, they were asked to describe their habitual diet before those changes. To obtain more accurate information, food scales and models from Nasco^®^ (Fort Atkinson, WI, USA), as well as household measures (e.g., cups and spoons), were used. These methods were intended to estimate the actual quantities of food consumed, as previously described [14,15]. Added sugar intake was defined as the consumption of all monosaccharides and disaccharides added during food processing, preparation, or at the table, as well as sugars naturally present in honey, syrups, and fruit juices. The percentage of added sugar intake was interpreted in accordance with World Health Organization guidelines, which recommend an intake of <5% of the total caloric intake [16]. Dietary n-3 intake was estimated as the sum of the following PUFAs: alpha-linolenic acid (18:3), stearidonic acid (18:4), eicosapentaenoic acid (20:5), docosapentaenoic acid (22:5), and docosahexaenoic acid (22:6). Dietary n-6 intake was calculated as the sum of linoleic acid (18:2) and arachidonic acid (20:4). The n-6:n-3 PUFA ratio was determined by dividing total n-6 intake by total n-3 intake (g/day). The cutoff value considered for adequate intake was ≤5:1 [17].

Micronutrient intake was compared with the reference values established by the National Institute of Medical Sciences and Nutrition “Salvador Zubirán” for the Mexican population [18], the NOM-051-SCFI/SSA1-2010 [19], and the DRI Dietary Reference Intakes: Applications in Dietary Assessment [20].

### 2.6. Biochemical Analysis

Blood samples were collected in the morning after an 8–10 h overnight fast by venipuncture using a BD Vacutainer^®^ system (Franklin Lakes, NJ, USA). Serum was separated by centrifugation at 4 °C for 15 min at 3500 rpm. Serum glucose, triglycerides (TG), total cholesterol (TC), and high-density lipoprotein cholesterol (HDL-c) were measured by dry chemistry using a Vitros 350 Analyzer (Ortho-Clinical Diagnostics, Johnson & Johnson Services Inc., Rochester, NY, USA). Low-density lipoprotein cholesterol (LDL-c) was calculated using the Friedewald formula when TG levels were <400 mg/dL [21]. The TG/HDL-c and TC/HDL-c ratios were calculated by dividing TG by HDL-c and TC by HDL-c, respectively.

### 2.7. Anthropometric Measurements

Anthropometric and body composition measurements were obtained after an 8–10 h overnight fast, with participants wearing light clothing and no shoes. Height was measured using a stadiometer (model 213, precision 0.1 cm, range up to 205 cm), and body weight was assessed using a calibrated digital scale (model 803, SECA GmbH & Co., Hamburg, Germany). BMI was calculated as weight (kg) divided by height squared (m^2^) [22]. WC was assessed following the ISAK protocol using a Lufkin Executive^®^ Thinline 2 mm measuring tape (New Brighton, MN, USA) [23], and abdominal circumference (AC) was measured at the most prominent part of the abdomen, using the umbilicus as a reference point. Body fat percentage (BF%) was determined by tetrapolar bioelectrical impedance analysis with the Quantum V Segmental (RJL Systems, Clinton Township, MI, USA), using an eight-point electrode configuration applied bilaterally to the hands and feet. Before electrode placement, the dorsal hand/wrist and foot/ankle sites were cleaned with alcohol pads. During the assessment, participants remained supine, and data were processed using the manufacturer’s software (RJL BC Segmental version 1.1.0) [24].

### 2.8. Statistical Analysis

Sample size was calculated using a formula for comparing two means with OpenEpi software version 3.0. The reference value was obtained from a study comparing dietary intake between normal weight and overweight Mexican adults [25]. A statistical power of 80% and a 95% confidence interval were used, resulting in a required sample size of 16 participants per study group.

All quantitative variables are presented as mean ± standard deviation (SD), while qualitative variables are expressed as absolute frequency (*n*) and percentage (%). For comparative analyses, participants were classified into CAD and non-CAD groups and stratified by sex. The Shapiro–Wilk test was used to assess the distribution of quantitative variables. When comparisons required adjustment for other variables, a general linear model was used. Categorical variables were compared using the chi-square test. All statistical analyses were conducted using SPSS version 25.0 (IBM Corp., Armonk, NY, USA). A two-tailed *p*-value < 0.05 was considered statistically significant.

### 2.9. Ethical Approval

All participants were informed about the research procedures, and written informed consent was obtained from those who agreed to participate. The study was conducted in accordance with the principles of the Declaration of Helsinki (2024). The protocol was approved by the Research and Biosecurity Ethics Committee of the Centro Universitario de Ciencias de la Salud (CUCS), Universidad de Guadalajara (Approval No. CI/08320).

## 3. Results

### 3.1. Characteristics of the Study Subjects

**Figure 1:**
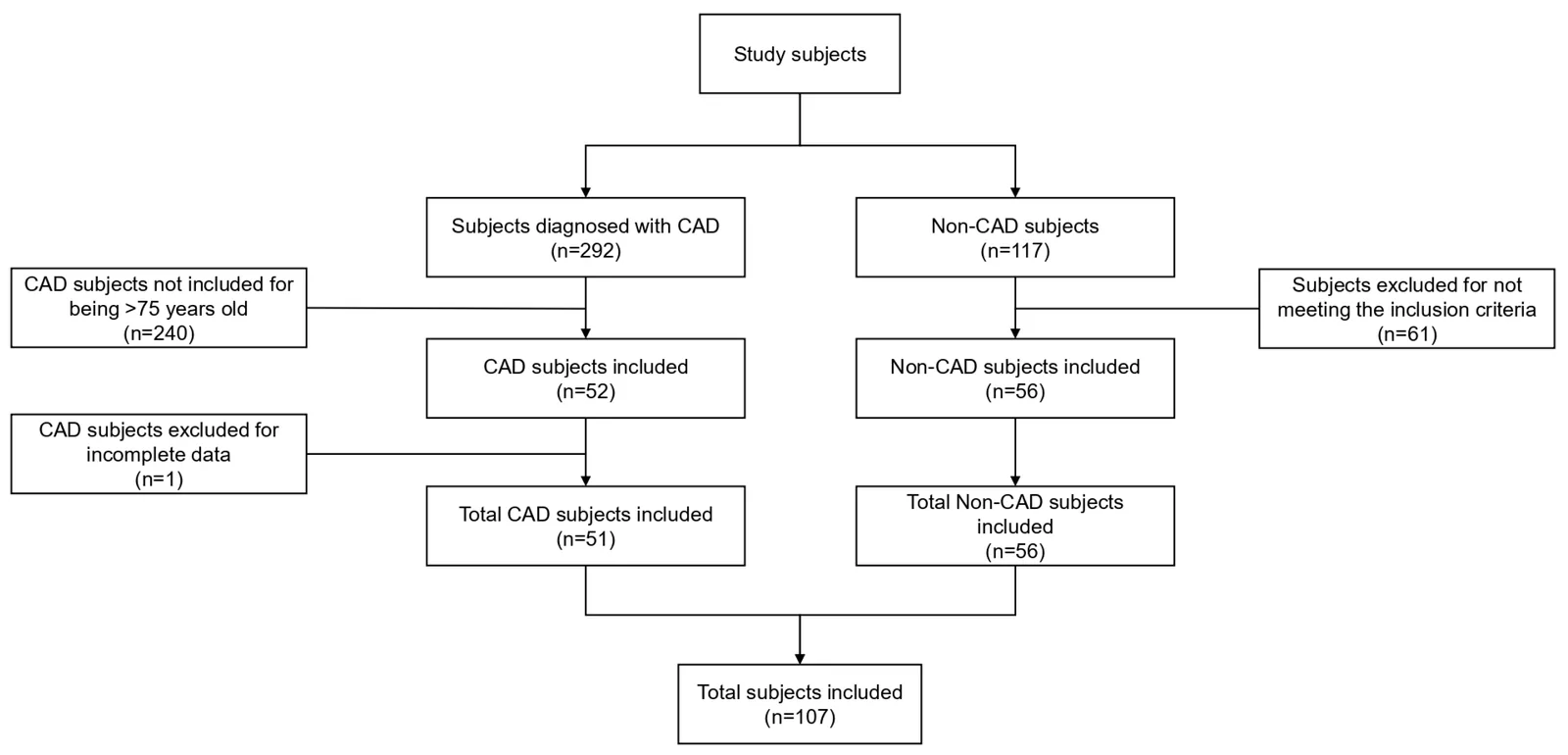
Flow diagram of the study subjects.

The study included 107 subjects (Figure 1), comprising 51 subjects with CAD and 56 non-CAD subjects. The CAD group consisted of 66.7% men (*n* = 34) and 33.3% women (*n* = 17), while the non-CAD group consisted of 60.7% men (*n* = 34) and 39.3% women (*n* = 22). Subjects were stratified by sex for comparative analyses. The mean age of women with CAD was 60.5 ± 12.1 years compared with 52.0 ± 12.4 years in non-CAD women (*p* = 0.039). Similarly, men with CAD were older than non-CAD men (60.0 ± 12.9 vs. 49.6 ± 10.2 years, *p* = 0.004).

### 3.2. Anthropometric and Biochemical Characteristics

Participants were stratified by sex for comparative analyses, as shown in Table 1. Regarding anthropometric variables, women with CAD exhibited a distinct body composition, with a tendency toward a higher body fat percentage (*p* = 0.051) and BMI (*p* = 0.054), as well as greater abdominal adiposity, as reflected by increased WC (*p* = 0.005) and AC (*p* = 0.028), compared with non-CAD subjects. No significant differences in anthropometric variables were found between men with or without CAD. Regarding the biochemical profile, CAD men had higher glucose levels than non-CAD men (*p* = 0.02). In non-CAD women, higher TC levels than in CAD women were found (*p* = 0.048). No significant differences were observed in other biochemical variables.

### 3.3. Macronutrient Intake

Macronutrient intake in CAD and non-CAD subjects according to sex is shown in Table 2. No significant differences in total energy intake or macronutrient distribution were observed between women with and without CAD. Likewise, no significant differences were found in the intake of specific fatty acids, dietary cholesterol, fiber, added sugar, or sodium; however, men with CAD showed a higher n-6:n-3 PUFA ratio intake (13.3 (10.8–15.8) in comparison to non-CAD men (8.5 (6–11), *p* = 0.012). In women, a tendency was found (CAD 12.5 (9.5–15.5) vs. non-CAD 8.6 (6–11.2), *p* = 0.063).

### 3.4. Micronutrient Intake

Micronutrient intake in CAD and non-CAD subjects according to sex is shown in Table 3. Mean intake of lycopene was lower in women with CAD compared to non-CAD women (*p* = 0.044).

Among men, participants with CAD also exhibited lower intake of several micronutrients compared with non-CAD men: vitamin A (*p* = 0.041), C (*p* = 0.003), folate (*p* = 0.035), potassium (*p* = 0.002), manganese (*p* = 0.036), copper (*p* = 0.006), iron (*p* = 0.016), beta-carotene (*p* = 0.044), lutein (*p* = 0.006), and lycopene (*p* = 0.009). Stigmasterol, campesterol, and betasitosterol intake were lower in non-CAD men (*p* = 0.002, 0.020, and 0.015, respectively).

Table 4 shows the percentage of participants who met the recommended intake for each micronutrient according to sex. Regarding adequate micronutrient intake, only 23.5% of women with CAD met the recommendations for vitamin K, compared with 59.1% of non-CAD women (OR 0.213, 95% CI, 0.052–0.869, *p* = 0.050). Although no other statistically significant differences were observed, women with CAD generally had lower percentages of adequate intake for several micronutrients, including vitamins A, C, and E, as well as calcium, potassium, manganese, selenium, and iron. Among men, adequate vitamin A intake was significantly less frequent in the CAD group than in the non-CAD group (35.3% vs. 50.0%, OR 0.360, 95% CI, 0.130–0.994, *p* = 0.046), whereas adequate selenium intake was significantly more frequent among participants with CAD (100% vs. 88.2%, OR 1.133 95% CI, 1.002–1.1.281, *p* = 0.039). No other statistically significant differences were identified between the groups.

When adequate micronutrient intake was compared between CAD and non-CAD subjects, participants with CAD had a significantly lower proportion of adequate intake of vitamin A (33.3% vs. 53.6%, OR 0.433 95% IC, 0.198–0.949, *p* = 0.035), vitamin K (19.6% vs. 42.9%, OR 0.325 95% CI, 0.136–0.777, *p* = 0.010), and vitamin C (45.1% vs. 64.3%, OR 0.456 95% CI, 0.210–0.992, *p* = 0.046) than non-CAD subjects. No significant differences were observed between other micronutrients; however, overall, participants with CAD show lower adequacy percentages for several micronutrients compared to non-CAD subjects (Table 5).

## 4. Discussion

CAD is a multifactorial condition that currently affects a substantial proportion of the Mexican population and represents a leading cause of death, accounting for 14.5% of deaths [26,27]. Its etiology is complex, and diet quality contributes significantly to disease development. In this regard, a dietary pattern characterized by a high intake of processed foods, added sugars, and red meats, together with a low intake of fruits, vegetables, whole grains, nuts, legumes, and fish, has been associated with the development, severity, and progression of CAD [28,29].

Although previous reports indicate that the Mexican population generally follows an inadequate diet [30], limited information is available regarding dietary patterns and micronutrient intake among individuals with CAD. Therefore, the main objective of this study was to analyze micronutrient intake in subjects with CAD compared to non-CAD subjects, given the established role of these nutrients in preventing lipoprotein oxidation and improving biochemical parameters, thereby contributing to protection against disease development and the prevention of clinical complications [31].

Regarding biochemical parameters, subjects with CAD had higher blood glucose levels than non-CAD subjects. This finding may be explained by the positive association between hyperglycemia and CAD, as hyperglycemia has been reported to alter endothelial nitric oxide synthase (eNOS) activity, thereby impairing nitric oxide (NO) production, which is essential for proper endothelial vasodilation. Moreover, hyperglycemia induces the production of reactive oxygen species (ROS), leading to endothelial dysfunction, increased expression of inflammatory cytokines, and, consequently, promoting a pro-inflammatory state [32,33].

In addition, certain trace elements, including magnesium, calcium, potassium, and zinc, are related to glycemic control [34]. In the present study, it was observed that more than 50% of women with CAD had a zinc intake below the recommended levels, which, according to previous reports, could contribute to the elevated glucose levels [35,36]. Zinc plays a role in insulin signaling through the activation of the phosphoinositide 3-kinase/protein kinase B (PI3K/Akt) pathway. Furthermore, insulin maturation and secretion require the formation of zinc–insulin hexamers; therefore, this micronutrient indirectly participates in glucose regulation [37].

The intake of certain types of lipids may also influence blood lipid concentrations. In the present study, men with CAD had a higher n-6:n-3 PUFA intake ratio than non-CAD men. This finding could be associated with the lower HDL-c concentrations observed in men with CAD, considering the report by Torres-Castillo et al., who found that individuals in the highest tertile of n-6:n-3 PUFA intake ratio (T3: >11.74:1) had lower HDL-c concentrations than those in the lowest tertile (T1: <8.39:1), although this difference was not statistically significant [8]. Similarly, Cartolano et al. reported an increase in HDL-c in subjects supplemented with 1800 mg of eicosapentaenoic acid and docosahexaenoic acid (EPA+DHA) for 8 weeks. Moreover, the HDL-c subfractions (HDL1, HDL2, and HDL3) increased to a greater extent than with n-6 PUFA supplementation [38]. The mechanism by which n-3 PUFAs influence HDL-c is not yet fully understood. However, n-3 PUFAs have been reported to regulate the expression of genes such as ATP-binding cassette transporter A1 (*ABCA1*) and apolipoprotein A-I (*ApoA-I*) [39]. In contrast, non-CAD men had higher intakes of dietary cholesterol compared to men with CAD, which could have contributed to the LDL-c concentrations observed in the non-CAD group [40]. These findings indicate that, although the non-CAD men had not experienced cardiovascular events, they were not free of cardiovascular risk factors, altered lipid profiles and higher intakes of dietary cholesterol.

Regarding the quantitative analysis of micronutrient intake, vitamin A intakes were significantly higher in non-CAD men. These differences could also be related to the HDL-c concentrations in the same study group, as suggested by Zalaket et al. and Gordon et al. [41,42]; vitamin A has been associated with increased levels of liver X receptor α (LXRα), a transcription factor that stimulates the expression of *ABCA1*, a protein that facilitates the transfer of free cholesterol and phospholipids to ApoA-I, leading to HDL-c formation [43,44,45]. Additionally, another micronutrient that has been associated with HDL-c concentrations is lycopene, by increasing the activity of lecithin–cholesterol acyltransferase (LCAT) and paraoxonase 1 (PON1). The effect on LCAT has been attributed to lycopene’s antioxidant activity as a free-radical scavenger, which may help preserve or enhance LCAT activity. In addition, increased PON1 activity has been attributed to lycopene’s role as a singlet oxygen quencher and its potential effects on oxidative-stress-related DNA methylation, thereby favoring *PON1* expression and activity [46,47].

Beta-carotene intake was also higher in non-CAD men. Beta-carotene is the main provitamin A carotenoid and has been described as a potent antioxidant, capable of scavenging free radicals derived from oxidized lipids in lipoproteins, thereby reducing lipid peroxyl radicals (LOO• to LOOH). This activity may be relevant to the prevention of cardiovascular disease because lipoprotein oxidation is a key process in atherosclerosis and, consequently, in CAD [48,49]. Another carotenoid analyzed was lutein, which was significantly higher in non-CAD men than in men with CAD. A meta-analysis showed that subjects with higher lutein intake had a lower risk of CAD (RR: 0.88, 95% CI: 0.80–0.98). This effect may be related to lutein binding to HDL-c via ApoA-I, thereby providing oxidative protection to the lipoprotein and enhancing its anti-atherogenic activity [50].

Regarding vitamin K, this study found a higher percentage of women in the non-CAD group with adequate intake than among women with CAD. Vitamin K has been associated with reduced cardiovascular risk through mechanisms involving the prevention of arterial calcification, modulation of inflammatory processes, and regulation of coagulation [51].

When the adequacy of micronutrient intake was analyzed according to sex, more than 50% of subjects had intakes below the recommended levels of vitamin D, vitamin E, biotin, pantothenic acid, and potassium. Inadequate micronutrient intake has been considered an important factor in CAD pathophysiology because these nutrients modulate processes involved in vascular damage and atherosclerotic progression [52].

Adequate vitamin D intake was uncommon in both groups, particularly among women with CAD. This finding is consistent with previous studies describing how vitamin D deficiency may impair the autophagic capacity of macrophages in response to oxidized LDL-c, thereby promoting foam cell formation. Vitamin D has been reported to inhibit foam cell formation through the vitamin D_3_–vitamin D receptor–tyrosine phosphatase SHP-1 (VitD3-VDR-PTPN6) pathway, which restores lipid autophagy in macrophages. Induction of PTPN6 inhibits the activation of signal transducer and activator of transcription 3 (STAT3), an autophagy inhibitor, thereby promoting the expression of autophagy-related proteins such as Beclin 1 (BECN1) and autophagy-related protein 5 (ATG5) [53].

Vitamin E intake was also particularly low among subjects with CAD in both sexes. This finding is relevant because alpha-tocopherol, an isoform of vitamin E, is a key antioxidant that inhibits lipid peroxidation, a critical process in atherosclerosis. Vitamin E deficiency reduces the neutralization of peroxyl and alkoxyl radicals, thereby promoting the oxidation of PUFAs in cell membranes and lipoproteins, endothelial dysfunction, and vascular injury. Alpha-tocopherol acts by transferring a hydrogen atom to lipid radicals, converting them into less reactive compounds, while the resulting alpha-tocopheroxyl radical can subsequently be reduced through reactions with other antioxidant molecules [54].

Regarding B vitamins, a low proportion of adequate intake of biotin and pantothenic acid was observed in both groups, irrespective of sex. Although evidence linking deficiencies of these vitamins to CAD is limited and no direct causal associations have been demonstrated in humans, both nutrients play important roles in key metabolic processes. Biotin acts as a cofactor for five carboxylases involved in cellular pathways such as gluconeogenesis, fatty acid synthesis, and branched-chain amino acid metabolism [55]. Similarly, pantothenic acid is essential for the biosynthesis of fatty acids, coenzyme A, cholesterol, and acetylcholine. However, deficiencies of these vitamins are uncommon, because they are widely distributed in a variety of foods [56].

Potassium intake adequacy was also low in both groups. Potassium plays an important role in blood pressure regulation by modulating ion channel activity and regulating vascular tone. Potassium deficiency has been associated with activation of the renin-angiotensin-aldosterone system and increased ROS production [57].

When magnesium intake was analyzed, more than 50% of non-CAD men were found to have inadequate intake. Magnesium acts as a calcium antagonist by competing for its binding sites in ion channels, thereby reducing Ca^2+^ overload and modulating catecholamine release. These effects contribute to endothelial vasodilation and NO availability. Magnesium deficiency has been associated with hypertension, increased catecholamine release, and atherosclerosis [58].

Among subjects with CAD, a percentage of intake adequacy less than 50% was observed regarding iron in men and folate in women, compared to non-CAD. Iron is essential for oxygen transport, and its deficiency may induce tissue hypoxia and alter NO homeostasis [59]. Likewise, it was observed that folate deficiency reduces the availability of 5-methyltetrahydrofolate, which may increase homocysteine levels and decrease NO synthesis, thereby contributing to impaired endothelial function [60].

Regardless of whether the analyses were stratified by sex or clinical condition, similar patterns of inadequate intake were observed for several micronutrients. One possible explanation is that both study groups have broadly similar dietary patterns, particularly in terms of macronutrient distribution.

In contrast, the non-CAD group had higher percentages of adequate intake of vitamins A, C, and K than the CAD group. These differences may reflect potentially relevant nutritional characteristics between groups. However, given the cross-sectional design of the study, these findings should not be interpreted as evidence that greater intake of these vitamins prevented the development of CAD.

Regarding vitamins A and C, fewer than 50% of CAD men had adequate intakes. This inadequate intake may have relevant molecular implications in the context of atherosclerosis, as vitamin A-derived retinoic acid (RA) participates in the regulation of genes involved in cholesterol homeostasis. RA exerts its effect by binding to retinoic acid receptors (RARs) and retinoid X receptors (RXRs), which regulate the expression of genes involved in lipid metabolism. In particular, activation of the RAR/RXR complex stimulates ABCA1 and ABCG1 via liver X receptors (LXR), promoting cholesterol efflux to HDL-c and reducing foam cell formation in macrophages [61].

Moreover, vitamin C may exert cardioprotective effects through the modulation of vascular inflammation and oxidative stress, both of which are central mechanisms in CAD development. Higher plasma concentrations of vitamin C have been associated with lower levels of high-sensitivity C-reactive protein (hs-CRP), a marker of systemic inflammation, that is associated with cardiovascular risk. Vitamin C has also been reported to reduce interleukin 6 (IL-6), a key pro-inflammatory cytokine involved in cardiovascular disease. In terms of oxidative stress, vitamin C may reduce malondialdehyde (MDA), a product of lipid peroxidation and a marker of oxidative damage [62].

## 5. Strengths and Limitations

One of the strengths of this study is the comparison of micronutrient intake between subjects with and without CAD, which provides evidence on micronutrient consumption patterns in the Mexican population where information is still limited and identifies aspects of the diet that could be improved through strategies that promote a high-quality diet. However, this study has some limitations. Dietary intake was assessed using a habitual-day food record, which could introduce some recall bias, although it provides a valid picture of intake at the time of diagnosis. Furthermore, the small number of women with CAD in the sex-stratified analyses, combined with the lack of correction for multiple comparisons, suggests that these results should be interpreted as hypothesis-generating for future large-scale confirmatory studies.

Future studies should include three-day dietary records as well as the inclusion of variables like socioeconomic status and other lifestyle variables related to CAD with a sex-specific sample size. Finally, although the non-CAD group was classified based on clinical criteria and the Rose Angina Questionnaire, the presence of subclinical atherosclerosis cannot be completely ruled out without the measure of other clinical variables.

## 6. Conclusions

A high prevalence of inadequate intake of key micronutrients, including vitamins A, C, and K, was found; however, these inadequacies were more pronounced among participants with CAD, especially in men who also showed more n-6:n-3 PUFA ratio intake. In addition, the intake of some antioxidant compounds such as lycopene, lutein, and beta-carotene were lower in CAD subjects. This dietary pattern can be associated with an Occidentalized diet and an obesogenic environment. Although the development of CAD cannot be directly attributed to micronutrient intake, it should be considered a potentially modifiable component of lifestyle, as an inadequate diet has been associated with unfavorable cardiometabolic profiles. These findings highlight that micronutrient consumption and overall dietary quality represent a relevant lifestyle factor in the prevention and management of CAD.

## Figures and Tables

**Table 1:** Comparison of anthropometric and biochemical variables between CAD and non-CAD subjects.

Variables	Women			Men		
	CAD *n* = 17	Non-CAD *n* = 22	*p*-Value	CAD *n* = 34	Non-CAD *n* = 34	*p*-Value
Anthropometrical						
Body fat (%)	41 (37.9–45)	35.9 (33–38.9)	0.051	28.9 (25.3–32.4)	29.1 (26–32.1)	0.939
BMI (kg/m^2^)	30.3 (27.8–32.9)	27 (24.8–29.1)	0.054	28.5 (26.4–30.7)	29.3 (27.1–31.4)	0.648
WC (cm)	95.1 (89.6–100.5)	84.3 (79.7–88.9)	0.005	98.2 (93.3–103.2)	98.7 (93.7–103.6)	0.906
AC (cm)	101.2 (94.9–107.5)	91.6 (86.3–96.9)	0.028	102.8 (97.7–107.8)	103.4 (98.3–108.4)	0.875
Biochemical						
Glucose (mg/dL)	108.8 (94.7–122.9)	91.2 (79.1–103.3)	0.136	125.7 (113.5–138)	99.8 (87.7–112)	0.02
TC (mg/dL)	146.5 (120–173)	190.1 (168.2–213)	0.048	152 (134.8–171)	166.3 (147–184.7)	0.416
TG (mg/dL)	125.9 (76.6–175.2)	180.5 (138–222.9)	0.183	180 (125–235)	156.7 (101–212)	0.639
HDL-c (mg/dL)	45.9 (32.6–59.2)	51 (39.7–62.6)	0.632	34.9 (30–39.5)	40.4 (35–45)	0.182
LDL-c (mg/dL)	75.2 (53.4–97)	103.7 (84.9–122.5)	0.118	83.8 (67– 101)	95.1 (78–112.3)	0.455
VLDL-c (mg/dL)	25 (15.2–35)	36.2 (27.7–44.7)	0.176	33.7 (23.7–43.7)	30.8 (20.7–41)	0.747
CT/HDL-c ratio	4 (3–4.9)	3.7 (2.9–4.6)	0.694	4.5 (3.9–5)	4.3 (3.7–4.9)	0.764
TG/HDL-c ratio	3.9 (2.2–5.7)	3.5 (2–4.9)	0.739	5.3 (3.8–6.8)	4.7 (3.2–6.2)	0.645

Values are presented as estimated mean and 95% CIs. The *p*-values were obtained using a general linear model test, adjusted by age. Biochemical data were also adjusted by BMI, comorbidities (hypertension and diabetes mellitus type 2), and medication use. AC: abdominal circumference; BMI: body mass index; WC: waist circumference; HDL-c: high density lipoprotein cholesterol; LDL-c: low density lipoprotein cholesterol; TC: total cholesterol; TG: triglycerides; VLDL-c: very low-density lipoprotein cholesterol.

**Table 2:** Comparison of macronutrient intake between CAD and non-CAD subjects.

Variables	Women		*p*-Value	Men		*p*-Value
	CAD *n* = 17	Non-CAD *n* = 22		CAD *n* = 34	Non-CAD *n* = 34	
Energy (kcal/day)	1849 ± 856	1831 ± 591	0.937	1925 ± 937	2183.9 ± 985	0.272
CH (%)	51.8 (45.4–58.1)	49.5 (44–55)	0.600	48.5 (43–53.7)	50.1 (45–55)	0.685
sCH (%)	83.7 (61.2–106.1)	91.7(72–111.2)	0.600	100.1 (75.2–125)	114.3 (89.3–139.2)	0.449
Ps (%)	18.2 (15.3–21)	16.4 (14–19)	0.373	18.4 (16.3–20)	18.4 (16.4–20.5)	0.968
TFA (%)	32 (27–38.7)	35 (30–40.1)	0.589	35.5 (31.1–40)	32.6 (28.2–37)	0.378
SFA (%)	10 (8.2–11.6)	9.8 (8.2–11.3)	0.917	10.5 (8.7–12.2)	10 (8.3–11.7)	0.744
MUFA (%)	10.1 (7.4–12.8)	13.2 (11–15.6)	0.098	12.1 (10.1–14)	11.6 (9.7–13.6)	0.775
PUFA (%)	8.8 (6.2–11.4)	8.4 (6.2–10.7)	0.827	9.2 (7.6–10.8)	6.3 (4.8–8)	0.019
Ratio n-6:n-3	12.5 (9.5–15.5)	8.6 (6–11.2)	0.063	13.3 (10.8–15.8)	8.5 (6–11)	0.012
Lauric acid (g)	0.4 (0.2–0.5)	0.3 (0.2–0.4)	0.459	0.5 (0.3–0.6)	0.5 (0.3–0.7)	0.591
Myristic acid (g)	1.5 (0.9–2)	1.2 (0.8–1.7)	0.454	2 (1.5–2.5)	1.8 (1.3–2.3)	0.601
Palmitic acid (g)	11 (8.5–13.5)	10.4 (8.2–12.5)	0.692	17 (14.2–20)	14.4 (11.5–17.3)	0.230
Stearic acid (g)	4.1(3–5)	4.4 (3–5)	0.923	7.3 (5.6–9)	6.2 (4.6–8)	0.391
Trans FA (g)	0.6 (0.3–0.8)	0.6 (0.3–0.7)	0.816	0.8 (0.4–1.2)	0.7 (0.3–1.1)	0.682
Cholesterol (mg)	291.4 (197–385.)	337 (255–419)	0.475	356.5 (274–439)	454 (372–537)	0.116
Total fiber (g)	29.6 (21.4–37.8)	30.8 (23.6–38)	0.835	30.5 (25–36)	33.3 (27.5–39)	0.531
Added sugar (g)	19.7 (6.1–33.4)	19.8 (8.3–31.2)	0.998	40.2 (22.6–57.8)	24.8 (7.5–42.1)	0.243
Sodium (mg)	1864 (1308–2420)	2090 (1605–2575)	0.549	2053 (1609–2497)	2449 (2006–2894)	0.237

Values are shown as mean ± SD, estimated mean, and 95% CIs. The *p*-value was obtained using a general linear model test, adjusted by age and total energy intake. kcal: kilocalories; CH: carbohydrates; sCH: simple carbohydrates (added sugar); Ps: protein; TFA: total fatty acids; SFA: saturated fatty acids; MUFA: monounsaturated fatty acids; PUFA: polyunsaturated fatty acids; n-6:n-3: omega 6 to omega 3 ratio; Trans FA: trans fatty acids.

**Table 3:** Comparison of micronutrient intake between CAD and non-CAD subjects.

Nutrient	Dietary Reference	Women		*p*-Value	Men		*p*-Value
		CAD *n* = 17	Non-CAD *n* = 22		CAD *n* = 34	Non-CAD *n* = 34	
Vitamin A (µg RAE) ^1^	W: 570 M: 730	990 (498–1481)	1051 (622–1480)	0.854	690 (293–1087)	1307 (911–1704)	0.041
Vitamin D (µg) ^2^	10	4.1 (2.6–5.5)	4.1 (2.8–5.3)	0.989	6.0 (4.4–7.6)	4.8 (3.2–6.4)	0.310
Vitamin E (mg) ^2^	11	7.7 (4.9–10.5)	10.1 (7.7–12.6)	0.207	8.1 (5.9–10.4)	9.4 (7.2–11.6)	0.448
Vitamin K (µg) ^1^	W: 75 M: 100	61.8 (16.2–139)	133.1 (65–201)	0.183	65.3 (9.5–121)	119.2 (63–175)	0.200
Vitamin C (mg) ^1^	W: 75 M: 84	104 (37–172)	159 (100–218)	0.241	70 (8.3–132)	215 (153–277)	0.003
Thiamin (mg) ^1^	W: 0.9 M: 1	1.2 (1.0–1.4)	1.2 (1.0–1.4)	0.715	1.5 (1.2–1.8)	1.8 (1.5–2.0)	0.205
Riboflavin (mg) ^1^	W: 0.9 M: 1	1.6 (1.2–2.0)	1.6 (1.3–1.9)	0.888	1.8 (1.5–2.0)	1.9 (1.7–2.2)	0.443
Niacin (mg) ^1^	W: 12 M: 13	17.3 (12.8–21.7)	16.8 (12.9–20.6)	0.866	22.0 (17.7–26.3)	24.0 (19.7–28.3)	0.533
Pantothenic acid (mg) ^1^	5	4.0 (3–5)	3.7 (2.8–4.5)	0.664	3.8 (2.9–4.7)	5.1 (4.2–6.0)	0.067
Pyridoxine (mg) ^1^	W: 1 M: 1.1	2.0 (1.6–2.5)	1.9 (1.5–2.3)	0.708	2.4 (1.9–2.8)	2.8 (2.3–3.2)	0.229
Biotin (µg) ^3^	30	11.1 (6.5–15.7)	16.4 (12.4–20.4)	0.094	12.8 (8.2–17.5)	15.4 (11.1–19.7)	0.452
Folate (µg) ^3^	320	356 (273–439)	361 (288–433)	0.928	329 (254–404)	449 (375–524)	0.035
Cobalamin (µg) ^2^	2.4	3.8 (2.9–4.6)	3.1 (2.3–3.8)	0.235	5.7 (3.8–7.7)	4.6 (2.6–6.5)	0.438
Calcium (mg) ^2^	900	759 (561–958)	770 (597–943)	0.935	893 (738–1047)	968 (813–1122)	0.518
Phosphorus (mg) ^2^	664	1416 (1224–1608)	1338 (1171–1506)	0.550	1885 (1684–2087)	1718 (1517–1920)	0.272
Potassium (mg) ^4^	3500	2959 (2322–3595)	3148 (2593–3704)	0.661	2895 (2411–3379)	4038 (3554–4522)	0.002
Magnesium (mg) ^1^	W: 260 M: 340	330 (267–393)	359 (304–414)	0.508	382 (322–443)	427 (366–487)	0.325
Manganese (mg) ^5^	W: 1.8 M: 2.3	2.0 (1.4–2.6)	2.1 (1.6–2.6)	0.780	1.9 (1.0–2.9)	3.5 (2.5–4.4)	0.036
Selenium (µg) ^2^	41	96.5 (70.4–122)	89.2 (66.4–111)	0.679	121 (104–139)	125 (107–142)	0.800
Copper (mg)		1.1 (0.8–1.3)	1.2 (1.0–1.4)	0.368	1.2 (1.0–1.4)	1.6 (1.4–1.8)	0.006
Zinc (mg) ^2^	10	10.2 (8.4–11.9)	9.3 (7.8–10.9)	0.498	14.0 (12.2–15.7)	13.0 (11.2–14.8)	0.484
Iron (mg) ^1^	W: 12 M: 15	13.6 (11.6–15.7)	14.0 (12.2–15.8)	0.793	15.1 (13.2–16.9)	18.5 (16.6–20.3)	0.016
Alpha carotene (µg)		1357 (456–2259)	1051 (264–1837)	0.616	784 (56.2–1512)	1273 (545–2001)	0.373
Beta carotene (µg)		4062 (1535–6589)	4842 (2637–7047)	0.649	2358 (419–4298)	5334 (3395–7274)	0.044
Beta cryptoxanthin (µg)		90.3 (31.7–212)	222 (116–329)	0.116	166 (0.32–332)	304 (138–470)	0.271
Lutein (µg)		1641 (392–3676)	3282 (1507–5057)	0.238	722 (83–1528)	2445 (1639–3250)	0.006
Lycopene (µg)		3989 (787–8766)	10,687 (6518–14,855)	0.044	2335(1998–6332)	10,775 (6441–15,109)	0.009
Betaine (mg)		4.2 (3.6–18.2)	20.8 (8.6–33)	0.087	15.5 (6.1–25)	17.5 (8.1–27)	0.779
Phytosterol (mg)		22.1 (8.1–36.1)	32.3 (20.1–44.5)	0.283	25.4 (11.6–39.3)	31.7 (18.8–44.6)	0.538
Stigmasterol (mg)		9.7 (4.7–14.8)	4.8 (0.7–8.9)	0.141	16.9 (10.4–23.3)	3.6 (1.8–7.3)	0.002
Campesterol (mg)		25.1 (7.0–43.2)	22.7 (7.8–37.5)	0.837	47.9 (26.5–69.4)	11.5 (6.7–29.8)	0.020
Betasitosterol (mg)		91.7 (38.3–145)	62.8 (19.1–106)	0.410	156 (87.9–225)	34.8 (23.7–93.4)	0.015

Data presented as estimated mean and 95% CIs. The *p*-values were obtained using a general linear model test, adjusted for age and total energy intake. W: women, M: men. Dietary reference values were based on the recommended daily intake established by the ^1^ National Institute of Medical Sciences and Nutrition Salvador Zubirán; ^2^ NOM-051-SCFI/SSA1-2010; ^3^ DRI Dietary Reference Intakes: Applications in Dietary Assessment; ^4^ World Health Organization. Potassium intake guidelines for adults and children; ^5^ National Institutes of Health Office of Dietary Supplements.

**Table 4:** Comparison of the percentages of CAD and non-CA subjects with adequate micronutrient intake, stratified by sex.

Nutrient	Women		*p*-Value	Men		*p*-Value
	CAD *n* = 17 % (*n*)	Non-CAD *n* = 22 % (*n*)		CAD *n* = 34 % (*n*)	Non-CAD *n* = 34 % (*n*)	
Vitamin A	52.9 (8)	59.1 (13)	0.455 ^a^	35.3 (9)	50 (17)	0.046 ^a^
Vitamin D	0 (0)	4.5 (1)	1.000 ^b^	23.5 (8)	5.8 (2)	0.083 ^b^
Vitamin E	23.5 (4)	31.8 (7)	0.725 ^b^	20.5 (7)	26.5 (9)	0.567 ^a^
Vitamin K	23.5 (4)	59.1 (13)	0.050 ^b^	17.6 (6)	32.3 (11)	0.161 ^a^
Vitamin C	52.9 (9)	72.7 (16)	0.201 ^a^	41.1 (14)	58.8 (20)	0.146 ^a^
Pantothenic acid	29.4 (5)	18.2 (4)	0.465 ^b^	32.3 (11)	41.2 (14)	0.451 ^a^
Niacin	52.9 (9)	68.2 (15)	0.332 ^a^	76.4 (26)	79.4 (27)	0.770 ^a^
Biotin	5.9 (1)	9.1 (2)	1.000 ^b^	8.8 (3)	14.7 (5)	0.713 ^b^
Folate	41.2 (7)	50 (11)	0.584 ^a^	55.8 (19)	50 (17)	0.627 ^a^
Calcium	23.5 (4)	27.3 (6)	1.000 ^b^	29.4 (10)	44.1 (15)	0.209 ^a^
Potassium	23.5 (4)	31.8 (7)	0.725 ^b^	41.2 (14)	41.2 (14)	1.000 ^a^
Magnesium	64.7 (11)	72.7 (16)	0.590 ^a^	52.9 (18)	44.1 (15)	0.467 ^a^
Manganese	41.2 (7)	68.2 (15)	0.092 ^a^	41.2 (14)	44.1 (15)	0.806 ^a^
Selenium	88.2 (15)	95.5 (21)	0.401 ^a^	100 (34)	88.2 (30)	0.039 ^a^
Zinc	41.2 (7)	40.9 (9)	0.987 ^a^	61.7 (21)	55.9 (19)	0.622 ^a^
Iron	58.8 (10)	63.6 (14)	0.759 ^a^	44.1 (15)	50 (17)	0.627 ^a^

Data presented as the frequency and percentage of subjects with adequate micronutrient intake according to dietary reference values. The ^a^ *p*-values were obtained using the chi-square test. ^b^ *p*-values were obtained using Fisher’s exact test.

**Table 5:** Comparison of adequate micronutrient intake between CAD and non-CAD subjects.

Nutrient	CAD *n* = 51 % (*n*)	Non-CAD *n* = 56 % (*n*)	*p*-Value
Vitamin A	33.3 (17)	53.6 (30)	0.035 ^a^
Vitamin D	15.7 (8)	5.4 (3)	0.112 ^b^
Vitamin E	21.6 (11)	28.6 (16)	0.405 ^a^
Vitamin K	19.6 (10)	42.9 (24)	0.010 ^a^
Vitamin C	45.1 (23)	64.3 (36)	0.046 ^a^
Pantothenic acid	31.4 (16)	32.1 (18)	0.932 ^a^
Niacin	68.6 (35)	75 (42)	0.464 ^a^
Biotin	8.5 (4)	12.5 (7)	0.750 ^b^
Folate	51 (26)	50 (28)	0.919 ^a^
Calcium	27.5 (14)	37.5 (21)	0.268 ^a^
Potassium	35.3 (18)	37.5 (21)	0.813 ^a^
Magnesium	56.9 (29)	55.4 (31)	0.875 ^a^
Manganese	41.1 (21)	53.5 (30)	0.200 ^a^
Selenium	96.1 (49)	91.1 (51)	0.181 ^a^
Zinc	54.9 (28)	50 (28)	0.612 ^a^
Iron	49 (25)	55.4 (31)	0.512 ^a^

Data presented as the frequency and percentage of subjects with adequate micronutrient intake according to dietary reference values. The ^a^ *p*-values were obtained using the chi-square test. ^b^ *p*-values were obtained using Fisher’s exact test.

## Data Availability

The data presented in this study are available from the corresponding author upon reasonable request, subject to privacy restrictions.

## Abbreviations

The following abbreviations are used in this manuscript:

ABCA1	ATP-binding cassette transporter A1
ABCG1	ATP-binding cassette sub-family G member 1
AC	Abdominal circumference
ApoA-1	Apolipoprotein A-1
BMI	Body mass index
CAD	Coronary artery disease
CH	Carbohydrate
CI	Confidence interval
DHA	Docosahexaenoic acid
DNA	Deoxyribonucleic acid
eNOS	Endothelial nitric oxide synthase
EPA	Eicosapentaenoic acid
HDL-c	High-density lipoprotein cholesterol
hs-CRP	High-sensitivity C-reactive protein
IL-6	Interleukin 6
LCAT	Lecithin–cholesterol acyltransferase
LDL-c	Low-density lipoprotein cholesterol
LXRα	Liver X receptor α
MDA	Malondialdehyde
MUFA	Monounsaturated fatty acids
NF-κB	Nuclear Factor kappa B
NO	Nitric oxide
n-3	Omega-3
n-6	Omega-6
PPARα	Peroxisome Proliferator-Activated Receptor Alpha
PPARγ	Peroxisome Proliferator-Activated Receptor Gamma
PON1	Paraoxonase 1
PUFA	Polyunsaturated fatty acids
RAR	Retinoic acid receptors
ROS	Reactive oxygen species
RXR	Retinoid X receptors
sCH	Simple carbohydrates (added sugar)
SD	Standard deviation
SFA	Saturated fatty acids
T2DM	Type 2 diabetes mellitus
TC	Total cholesterol
TFA	Total fatty acids
TG	Triglycerides
Trans FA	Trans fatty acids
VLDL-c	Very low-density lipoprotein cholesterol
WC	Waist circumference
